# Tailoring of organic dyes with oxidoreductive compounds to obtain photocyclic radical generator systems exhibiting photocatalytic behavior

**DOI:** 10.3762/bjoc.10.92

**Published:** 2014-04-25

**Authors:** Christian Ley, Julien Christmann, Ahmad Ibrahim, Luciano H Di Stefano, Xavier Allonas

**Affiliations:** 1 Laboratory of Macromolecular Photochemistry and Engineering, University of Haute Alsace, 3b rue Alfred Werner, 68093 Mulhouse, France

**Keywords:** computation, electron transfer, kinetic, photocatalysis, photochemistry, photocyclic initiating system, photopolymerization, photoredox catalysis, radical generator

## Abstract

The combination of a dye which absorbs the photon, an electron acceptor and an electron donor leading to energy conversion through electron transfer, was the basis of the so called three-component systems. In this paper, an experimental work combining Rose bengal dye with a triazine derivative as electron acceptor and ethyl 4-(dimethylamino)benzoate as electron donor, will underline the benefit of the photocyclic behavior of three-component systems leading to the dye regeneration. A thermodynamic approach of the photocycle is presented, followed by a mechanistic and computational study of ideal photocycles, in order to outline the specific kinetics occuring in so called photocatalytic systems. The simple kinetic model used is enough to outline the benefit of the cyclic system and to give the basic requirements in term of chemical combination needed to be fulfilled in order to obtain a photocatalytic behavior.

## Introduction

Among the possible usage of light, the conversion of photons into chemical energy, as stored into radicals or ions, is of great interest. As a part of this research area, the development of photoradical generators (PRG) is still a lively topic that finds applications in triggering bioactivity [1], drug or fragrance release [2–4], microelectronics [5], water catalysis reduction [6–8], and laser imaging [9]. In organic chemistry, the development of new methods for organic synthesis was achieved by use of photolabile protective groups, which could be released under irradiation by light [10–11]. Since many decades in industry, PRG are used in photopolymerization, a field in which the PRG prompt the initiation of the polymerization through a chain reaction [12]. Photopolymerization was first used over 4000 years ago in the mummification process [13]. During the last decades the number of commercial applications is still continuously increasing. By example, photopolymer applications are found in electronic materials [14], printing materials [15], optical and electro-optical materials [16–17], fabrication of devices and materials [18], adhesives and sealants [19], coatings [20] and surface modifications [21–22].

**Scheme 1:**
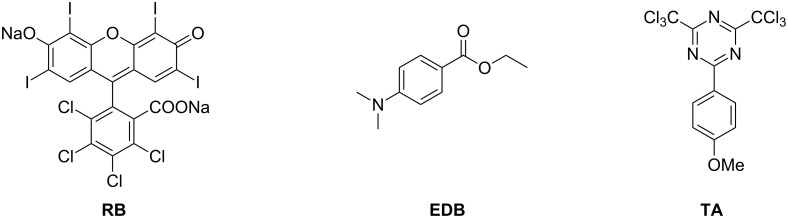
Chemical structures of RB, EDB and TA.

The great interest in PRG application to free radical polymerization (FRP) has led to the development of two major classes of photoinitiating systems (PIS): Type I and Type II. In Type I PIS, the excited states reached after light absorption undergo a cleavage leading to the production of two initiating radicals [5,23–24]. However, most Type I PIS are only active under UV–blue irradiation [25–26]. To overcome this spectral limitation, Type II PIS were developed. They contain the photosensitizer (PS) which absorbs the light and a coinitiator (Co) which reacts with PS excited states through hydrogen abstraction or electron transfer reaction (see Scheme 1). Numerous dyes were reported as PS [9,27–32]. Hydrogen donor coinitiators could be amines [33–38], ethers [39–40], sulfides [41–43] or thiols [43–45]. Electron transfer coinitiators could be borate salts [46–47], iodonium [48–49] or triazine [32] derivatives. However, if Type II PIS gain sensitivity in the visible part of the electromagnetic spectrum, their efficiency is lower than Type I PIS. To gain more reactivity the so-called three component systems (3-cpt) were developed by adding a redox additive to Type II systems [32,50–52]. A higher yield of initiating radicals is generally claimed in such cases. Moreover, the dye is recovered during the process and is newly available to absorb light, running into a new cycle [9,32]. These photocyclic initiating systems (PCIS), should then present a somehow constant absorbance, leading to constant and efficient absorption of the incident photons. Both features are responsible for the higher efficiency of PCIS [9,31,53–55] in photopolymerization reactions. Therefore, as the dye is regenerated during the photochemical reaction, a catalytic behavior appears, leading to the so-called photocatalytic system.

In this paper an experimental and mechanistic study of Type II PIS will be given and compared with a PCIS. Then, in order to improve the knowledge of PCIS, a thermodynamic and mechanistic approach of PCIS exhibiting an ideal photocatalytic behavior will be presented. The proposed scheme will be used as model to run some computation. This will permit to compare and discuss the advantages, the specificity of this kind of photocyclic systems and outline the important features and conditions which have to be fulfilled in order to obtain high performances for this kind of photocatalytic systems. This ideal approach will permit a better general understanding of the complex kinetics underlying PCIS chemical reactions, allowing a better and simplest selection of chemicals combination.

## Results and Discussion

In order to outline this behavior, the photochemical consumption of the dye (i.e., photolysis) was studied in a typical Type II and a 3-cpt PCIS based on the Rose Bengal as dye, and a triazine derivative (TA) as an acceptor (coinitiator). In addition, an amine (ethyl 4-(dimethylamino)benzoate, EDB) was chosen as redox (electron donor) additive for the PCIS (see Scheme 1).

### Type II photoinitiating system

**Scheme 2:**
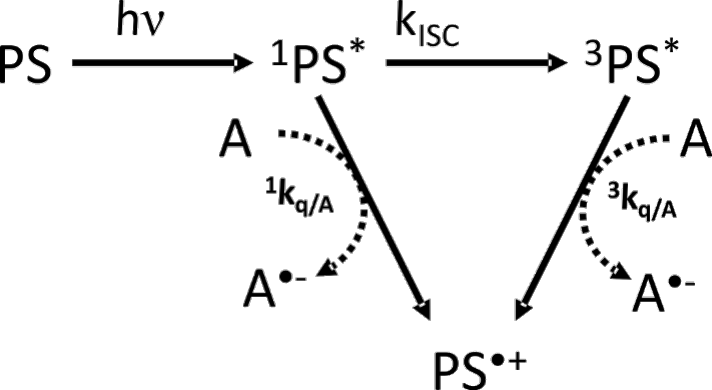
Type II PIS mechanisms. PS: photosensitizer; ^1,3^PS^*^: singlet and triplet PS excited states; PS^•+^: oxidized PS; A: electron acceptor coinitiator; A^•−^: reduced electron acceptor.

In Scheme 2, the typical reaction mechanism of a dye (PS) with an electron acceptor (A) is depicted. After absorption of actinic light (*h*ν), the PS reaches its singlet excited state (^1^PS^*^) and after intersystem crossing (*k*_ISC_), its triplet excited state (^3^PS^*^). From both these excited states, an electron transfer can occur from the PS to A (with quenching rate constants ^1^*k*_q/A_ and ^3^*k*_q/A_, respectively). The oxidized form of the dye (PS^•+^) is formed together with the reduced acceptor (A^•−^) which lead to initiating radicals [33,48–49].

As a consequence, the PS is consumed (i.e., photolysed or bleached) during the photoinduced electron transfer reaction. Thus, according to Beer–Lambert's law, the absorbance of the system decreases during the reaction: this bleaching could be followed by UV–visible absorption spectroscopy.

**Figure 1:**
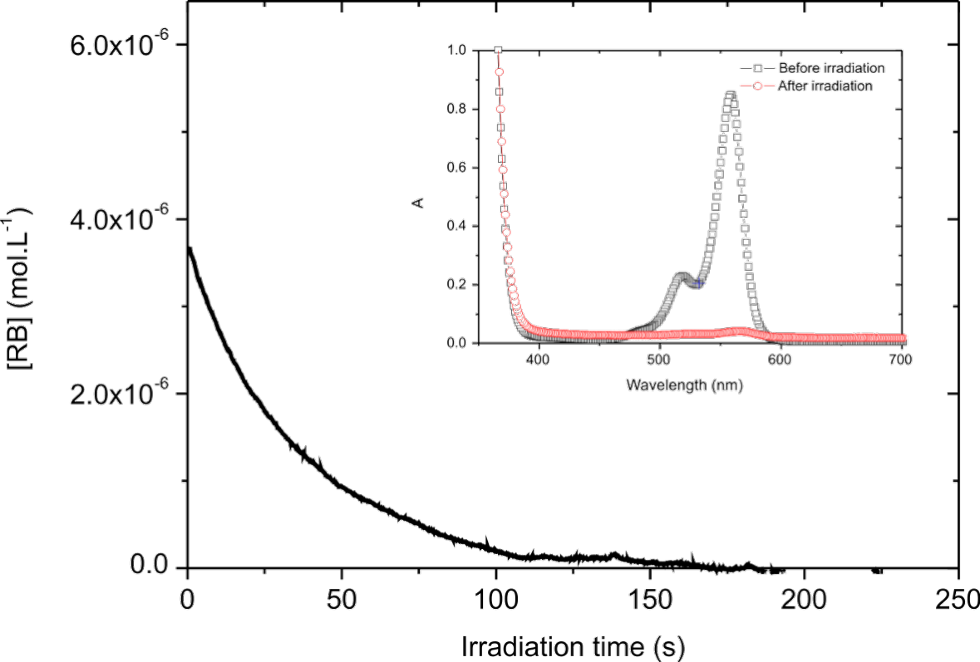
Evolution of RB concentration as a function of irradiation time (λ = 532 nm, 9 mW·cm^−3^); insert: absorption spectra obtained before and after irradiation.

The photolysis of an acetonitrile solution of RB/TA was done within a 1 cm width cell with a monochromatic 532 nm laser diode tuned to 9 mW/cm^2^ intensity. This will correspond to the 9 mW/cm^3^ computation condition (vide infra). The initial concentration was [RB]_0_ = 6.5 10^−5^ mol·L^−1^ (with ε = 31900 M^−1^·cm^−1^ and a length of l = 1 cm, this corresponds to an initial absorbance of around 0.2 at 532 nm) and [TA]_0_ = 10^−3^ mol·L^−1^. The transmitted laser diode light was real-time recorded. It is thus possible to calculate the optical density *A*(*t*) of the sample, which is then converted into [RB](*t*). It can be seen from Figure 1 that a fast decrease of [RB](*t*) occurs, so fast that the very beginning is not resolved by the detector. This indicates that the dye is consumed during irradiation, due to the electron transfer to TA. In about 100 s, the absorbance is almost zero indicating a complete consumption of the dye. As a consequence, the absorption spectrum completely vanishes after 5 min of irradiation, confirming the disappearance of the dye (see insert Figure 1). It should be noted that in the same conditions, no photolysis occurs for an acetonitrile solution of neat RB.

To quantify the consumption of the PS, the photolysis quantum yield (Φ_photolysis_) was determined. Φ_photolysis_ is defined by the ratio of the initial number of photosensitizer (PS) molecules present (*N*_PS_) to the total number of absorbed photons (*N*_abs_). It could be expressed by the following equation:

[1]
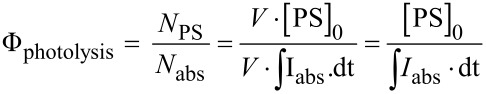


where *V* is the volume of the irradiated solution. The absorbed photon concentration is given by:

[2]
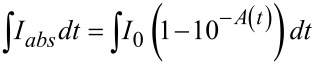


where *I*_0_ is the incident light intensity on the cell (9 mW·cm^−3^, i.e., 4·10^−5^ einstein·s^−1^·cm^−3^). The experimental measure of the absorbance *A*(*t*) allows the calculation of *I*_abs_(*t*) and a numerical integration. Accordingly, the total concentration of absorbed photon is calculated as 3.43·10^−4^ mol·L^−1^ for RB/TA. It should be noted here that the missing first 1 second of the fast decay of *A*(*t*) will represent, at maximum, 1.47·10^−5^ mol·L^−1^ of absorbed photons (by assuming a constant *A*(*t*) = 0.2 during this 1 s). This overestimation represents less than 5% of the total absorbed photons and could be neglected. Then, dividing the initial RB concentration by these values, a photolysis quantum yield of 0.19 for RB/TA is obtained.

### Photocyclic initiating system

**Scheme 3:**
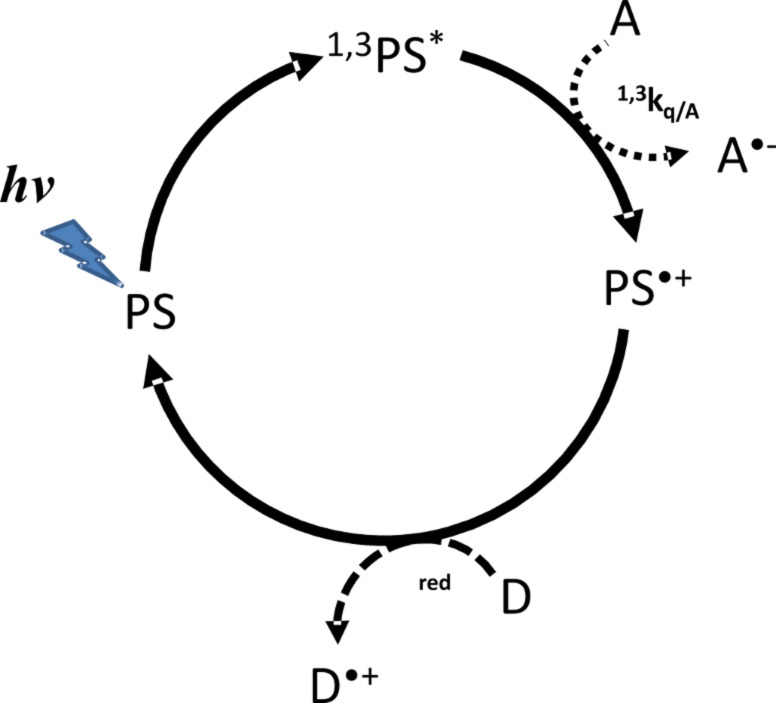
Photocatalytic behavior occurring in three component PIS. PS: photosensitizer; ^1,3^PS^*^: singlet and triplet PS excited states; A: electron acceptor; A^•−^: reduced form of acceptor; D: electron donor and D^•+^: oxidized donor.

A typical photocyclic initiating system (PCIS) consists of a light absorbing dye (PS), an electron acceptor (A) and an electron donor (D) (Scheme 3). In such systems, upon irradiation photoinduced electron transfer reaction between the dye and one of the components, for example A in Scheme 3, gives rise to a radical anion (A^•−^) and the oxidized PS (PS^•+^). Then, PS^•+^ can react with the electron donor D (*k*_red_) to regenerate the PS ground state (photocyclic reaction), leading to the formation of one radical cation D^•+^. Generally, A^•−^ and D^•+^ will give rise to initiating radicals for free radical polymerization, to hydrogen by water reduction or oxygen by water oxidation [6–8], etc. In an ideal case, a photocatalytic behavior is ensured when there is enough redox donor to make the dye surviving during a long period of time. It will be shown below that this behavior has great advantages; the most immediate is the fact that the absorbance of the PCIS is kept constant.

**Figure 2:**
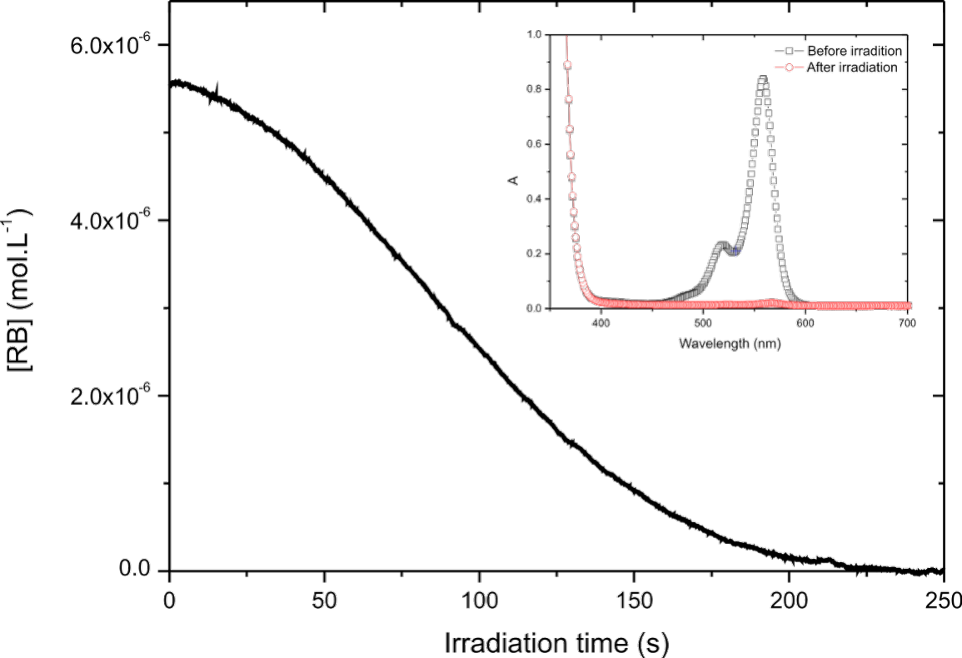
Evolution of [RB](*t*) in the photocyclic system RB/TA/EDB as a function of irradiation time (λ = 532 nm, 9 mW·cm^−3^). Insert: absorption spectra obtained before and after irradiation.

In order to exemplify the behavior of the photocycle involving the dye, the acceptor and the donor, the absorbance of a RB/TA/EDB solution was monitored during irradiation as for the Type II PIS. EDB was chosen as electron donor for the PCIS, because its reactivity toward ^3^PS is low compared to that of TA (vide infra). This ensures that ^3^PS will react mainly with TA in an oxidative cycle as proposed in Scheme 3. The solution was irradiated with the same laser diode and same output power. The same initial concentration of RB and TA was realized, and [EDB]_0_ was fixed to 10^−3^ mol·L^−1^. The experimental evolution of the [RB(t)] could be seen on Figure 2.

Compared to the Type II PIS, the behavior is quite different: the absorbance decreases very slowly at the beginning of the process, being almost constant for a couple of seconds. Then, the absorbance slowly decreases due to an increase of the photolysis rate with irradiation time. After 250 seconds, the residual absorbance is zero: in the photocyclic system, the time required for complete consumption of the dye is two times longer than in Type II PIS. This confirms the dye regeneration within a photocycle exhibiting a photocatalytic behavior.

The longer experimental surviving time of RB in the PCIS is in qualitative agreement with the proposed schemes. To quantify the cyclic behavior and to compare clearly Type II and PCIS, the quantum yield of dye photolysis (Φ_photolysis_) was also determined for RB/TA/EDB. The total concentration of absorbed photons for the PCIS is 0.0139 mol·L^−1^. Dividing the initial RB concentration by this value leads to a photolysis quantum yield of 4.7·10^−3^. This extremely low photolysis quantum yield obtained for the PCIS means that more than 210 photons are needed to bleach one dye molecule. The turnover number becomes very high for the PCIS while for the Type II system, only 5 photons are needed to bleach RB. This clearly demonstrates the cyclic regeneration of the dye in the selected three-component combination presented here. As a conclusion, these experimental results clearly confirm the photocyclic behavior of the selected 3-cpt system RB/TA/EDB. However, to get more insight and to understand the benefits of this process, a better understanding of the thermodynamics and the kinetics is necessary.

### Thermodynamics of the PCIS

**Scheme 4:**
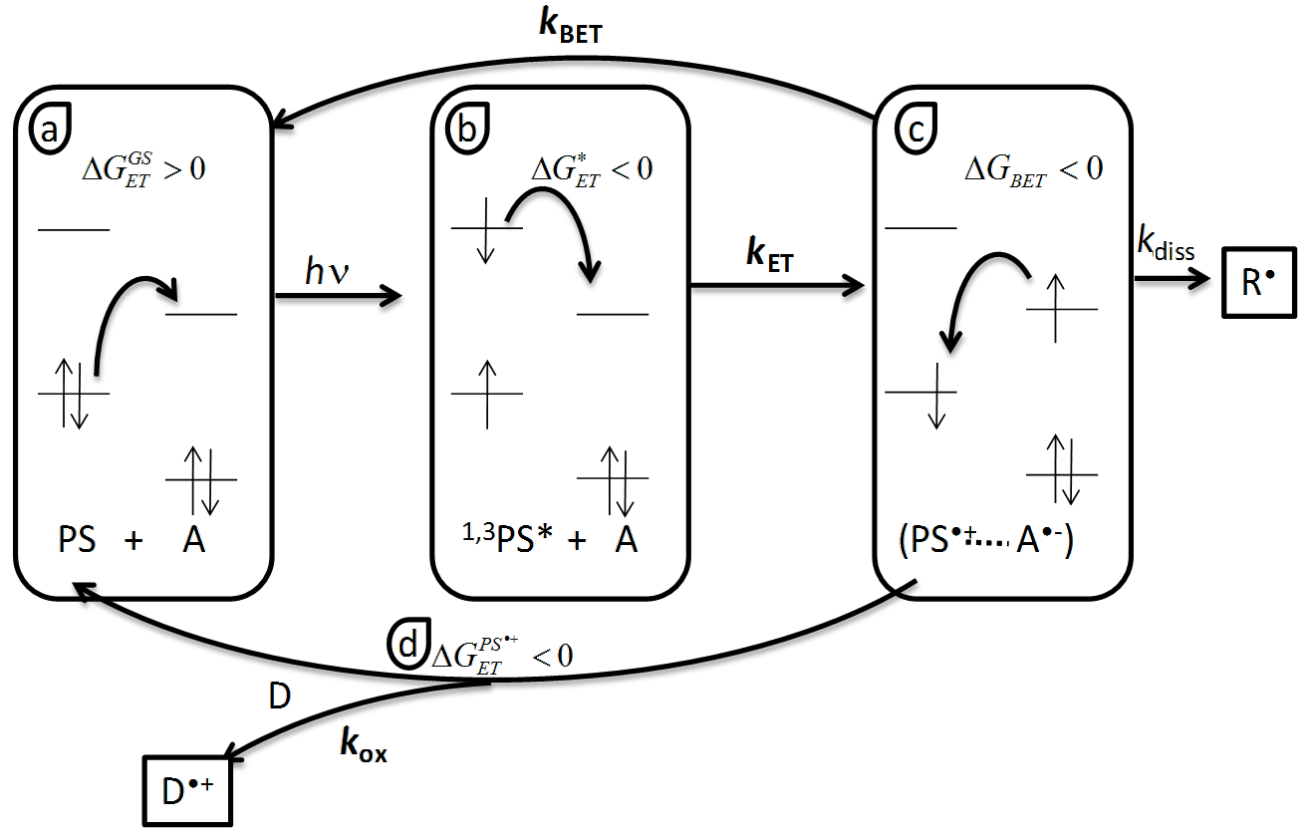
Thermodynamics of an oxidative three components PCIS, a) ground state reaction (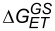), b) excited state reaction (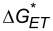), c) back electron transfer (BET, Δ*G_BET_*), d) PS regeneration (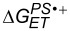).

Obviously, every mixture of an acceptor, a donor and a dye, would not give rise to a photocatalytic behavior. The components should be selected with care in order to get a cyclic behavior instead of competitive parallel reactions [54] where A and D compete to react with the PS excited states. Thus, a thermodynamic approach of the PCIS should help the selection of the candidates. Scheme 4 represents the different electron transfer reaction occurring in the oxidative PCIS according to the mechanism given in Scheme 3.

The first reaction is the electron transfer reaction between the dye ground state and the coinitiator. Its reactivity is governed by the corresponding Gibbs free energy change 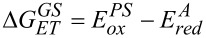 where *E*_ox_ and *E*_red_ are the half-wave oxidation and reduction potentials for the donor and the acceptor, respectively. 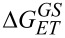 must be as high as possible to prevent any dark reaction.

After absorption of light the PS goes into singlet or triplet excited states, in which it becomes both more oxidant and more reducer. As a consequence, electron transfer reaction can occur with the acceptor A (Scheme 3). The reaction must be as much exergonic as possible. The values of the Gibbs free energy change 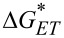 for photoinduced electron transfer is given by the Rehm–Weller equation [56]: 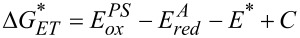, where E^*^ stands for the energy of the excited state. The Coulombic term C is usually neglected in polar solvent. 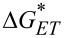 will determine the rate of electron transfer and the dye and the electron acceptor must be chosen such that 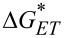 is negative enough to obtain a high electron transfer rate constant.

The third electron transfer step is the unwanted back electron transfer BET within the contact ion pair (PS^•+^···A^•−^) which leads to initial reactants. This one is the more tricky to handle. Δ*G_BET_* is given by 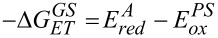. This reaction is generally quite exergonic and the rate compete with the dissociation rate *k*_diss_ of the contact ion pair into free solvated species. In practice BET reduce the overall radical generation quantum yields. Some new approaches allow to trigger this reaction [57–58].

The last important step is the dye regeneration. In order for the cycle and for the catalytic behavior to occur, the corresponding Gibbs free energy change 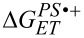 should be negative. If one assumes that the reduction potential of PS^•+^ is given by the oxidation potential of PS, then 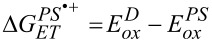. Thus, in order to achieve an efficient dye regeneration, the choice of the donor D should be carefully done to obtain an exergonic reduction of the oxidized PS.

This approach is also valid to a reductive catalytic cycle where the PS first reacts with the donor D, and then its reduced form is oxidized by the acceptor A. Table 1 summarizes the Gibbs free energy formula for estimation and the potential effect on the photocyclic behavior. The "Wanted values" row gives the values which must be obtained in order to get a possible photocyclic behavior of the selected components. The "Control" row summarizes the effect of the corresponding electron transfer on the PCIS final properties like shelf life, radical quantum yield and dye regeneration. One should also note that as 
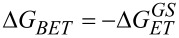
 it is always negative even if a positive value is wanted.

**Table 1:** Gibbs free energy of the different electron transfer reactions occurring in a three component PCIS.

Gibbs free energy	Wanted values	Control

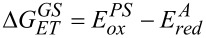	>0	Shelf life
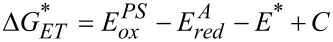	<0	Rate of electron transfer: radical quantum yields
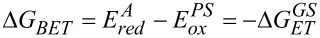	>0	Control of back electron transfer: radical quantum yields
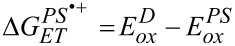	<0	Regeneration of the PS: photolysis quantum yield

### Computational studies of PCIS

**Scheme 5:**
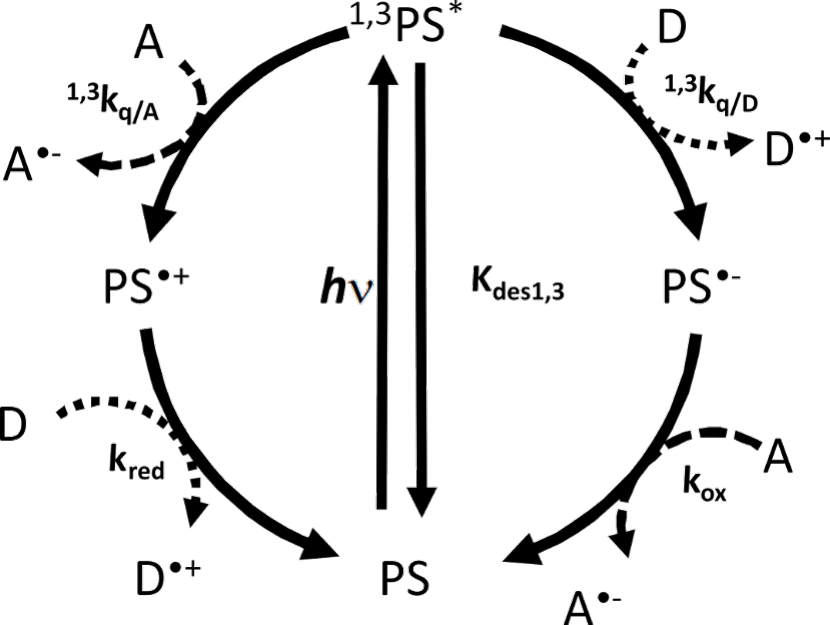
General photocatalytic cycle occurring in three components photocyclic systems. Two cycles are in competition: on the right side the first reaction occurs with the donor D defining the reductive pathway, while on the left side the primary reaction occurs with the acceptor A leading to the oxidative pathway.

As seen before, there are four-electron transfer reactions to manage in order to get a working PCIS. Moreover the full mechanistic description becomes more complicate by taking into account that both the oxidative and reductive pathways can be in competition (see Scheme 5). In order to get more insights into the description and comprehension of PCIS, a complete simulation of the photocyclic behavior of RB/TA/EDB was performed, comprising both a reductive and oxidative pathway (Scheme 5).

**Figure 3:**
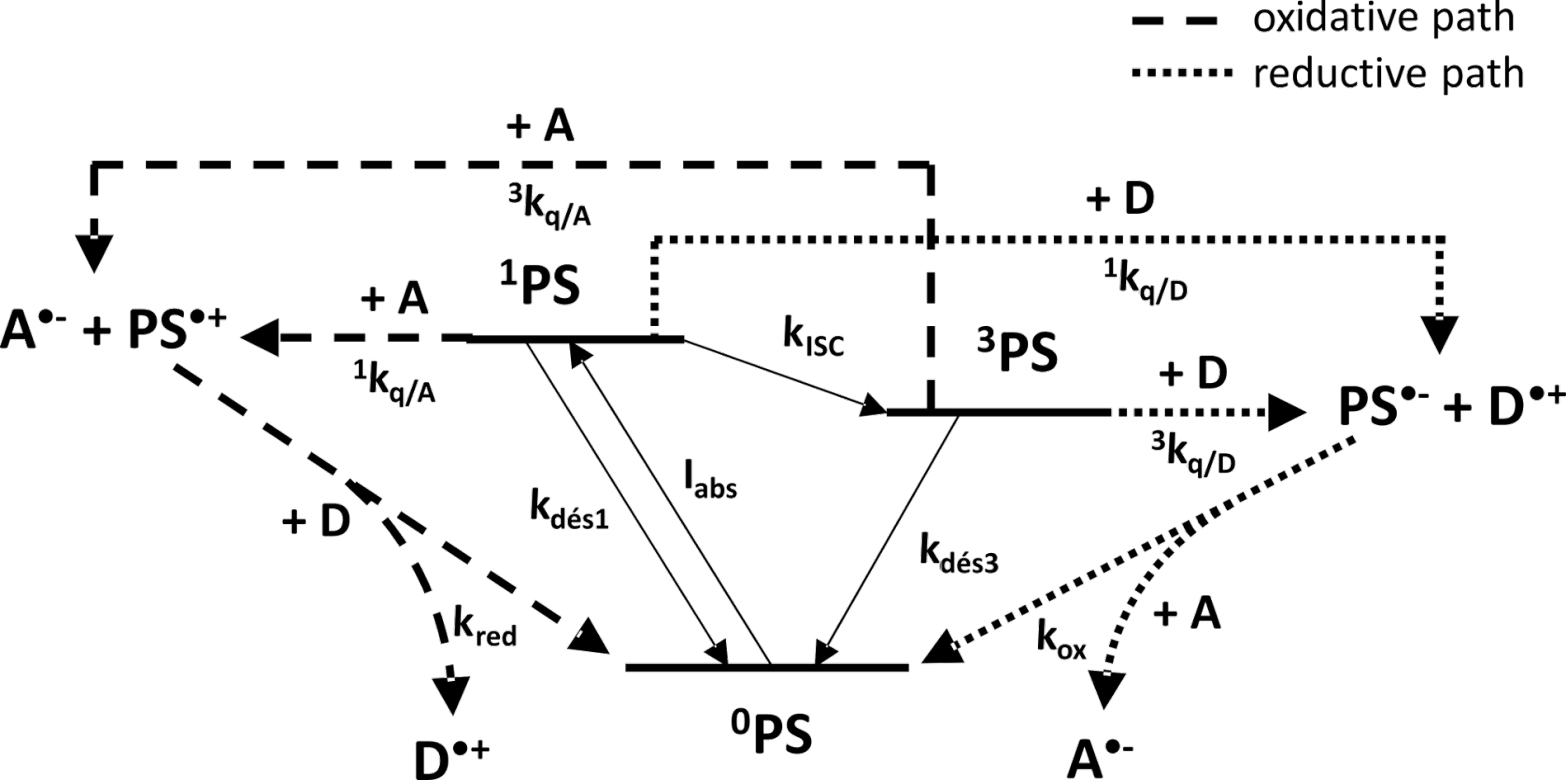
Mechanistic description of photocyclic system involved in the RB/TA/EDB system. The rate constants are defined in the text.

The full kinetic description of the reactions occurring in the complete cycle is displayed in Figure 3, where *I_abs_* is the number of absorbed photons per second; *k*_des1_, *k*_des3_ are the rate constants of singlet and triplet state deactivation to the ground state, respectively; *k*_ISC_ the rate constant of the intersystem crossing from ^1^PS to ^3^PS; ^1^*k*_q/A_, ^3^*k*_q/A_, (^1^*k*_q/D_, ^3^*k*_q/D_) the bimolecular electron transfer rate constant of the singlet and triplet excited state of the PS by the acceptor A (donor D), respectively; *k*_red_ and *k*_ox_ are the rate constants of reduction and oxidation of PS^•+^ and PS^•−^, respectively.

The time evolution of ground state PS ^0^PS, ^1^PS ^3^PS excited state, PS^•+^, PS^•−^, D, A, D^•+^ and A^•−^ are given by the following equations:

[3]
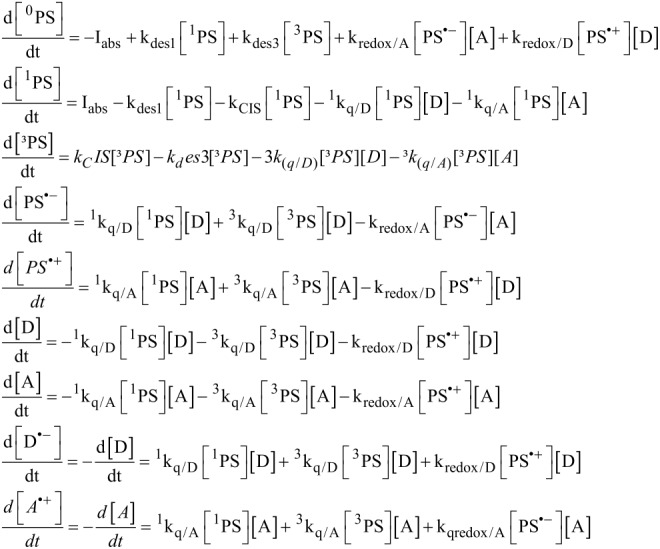


The absorption of the light is given by:


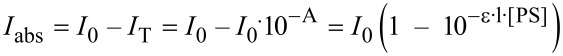


where I_0_ and I_T_ are the incident and transmitted light intensity on/through the sample respectively (einstein^.^L^−1.^s^−1^), ε is the molar extinction coefficient of the PS at the irradiation wavelength and l is the cell thickness.

### Simulation parameters

In order to simulate the photocyclic behavior, the different rate constants involved in the proposed mechanism were measured by time resolved spectroscopies (laser flash photolysis for triplet excited states and time correlated single photon counting for excited singlet states). The experimental quenching rate constants and their corresponding Δ*G*_ET_ are given in Table 2. As *k*_red_ and *k*_ox_ were not measurable, a value of 2·10^3^ M^−1^·s^−1^ was taken to perform the computations. This is justified by the fact that the radical recombination in the type II systems observed by laser flash photolysis occurs in the ms timescale. The last row of Table 2 contains the calculated Gibbs free energy of the dye regeneration redox reaction. These slightly endergonic values (0.42 and 0.12 eV) support the low *k*_red_ and *k*_ox_ rate constants used for computation.

**Table 2:** Thermodynamic and kinetic parameters of the PCIS simulation, *k*_q_: quenching rate constant of excited states.

Quencher		Δ*G*_ET_ (eV)/*k*_q_ (M^−1^·s^−1^)			
	^0^RB	^1^RB	^3^RB	RB^•+^	RB^•−^

EDB	2.07	−0.10/9.0 10^8^	0.27/4.50 10^4^	0.42/–	
TA	1.77	−0.40/6.0 10^9^	−0.03/1.7 10^7^		0.12/–

The measured electron transfer rate constants are in line with the ΔG, confirming the low reactivity of ^3^PS toward EDB. Intersystem crossing rate constant *k*_ISC_ was obtained from the triplet state quantum yield and singlet state lifetime according to [59]:


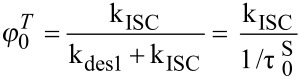


which leads to:


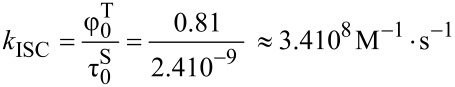


and to


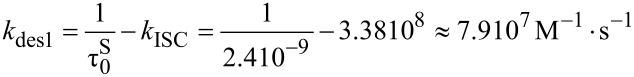


At this RB concentration, and in the absence of quencher, the triplet state lifetime was measured around 80 µs, leading to *k*_des3_= 1.25·10^4^ M^−1^s^−1^. The following parameters were used to perform the computation: the continuous incident light intensity *I*_0_ was fixed to 9 mW·cm^−2^ at 532 nm (i.e., 4·10^−5^ einstein·L^−1^·s^−1^). The initial PS (i.e., RB) concentration was fixed to [RB]_0_ = 6.50·10^−6^ M, with ε = 31900 M^−1^·cm^−1^ and a cell length of 1 cm this corresponds to an absorbance of 0.2 at 532 nm, in line with the experiments. The initial concentration of the quenchers were fixed at [TA]_0_ = 10^−2^ M and [EDB]_0_ = 10^−3^ M for TA (acceptor A) and EDB (donor D).

### Photophysical cycle

One must keep in mind that in PCIS, two cycles can occur: the photochemical one which involves the reaction of the dye excited states with chemical reactants, and an internal photophysical. This later is an energy waste, comprising the absorption of light, the deactivation by internal conversion and fluorescence of ^1^PS to the ground state ^0^PS, the intersystem crossing to ^3^PS, and the deactivation of the ^3^PS to ^0^PS. In order to achieve high quantum yield for the conversion of the light energy, this photophysical cycle should be avoided as much as possible. This means that the excited states must live as long as possible, the quenching rate constants should be as high as possible (exergonic reaction with high ^1,3^*k*_q/A_ and ^1,3^*k*_q/D_), and a high concentration of D and A should be used (high pseudo first order rate constants ^1,3^*k*_q/A_[A] and ^1,3^*k*_q/D_[D]). This will prevent the probability of natural excited state deactivation to be high.

### Simulation of Type II photoinitiating system

**Figure 4:**
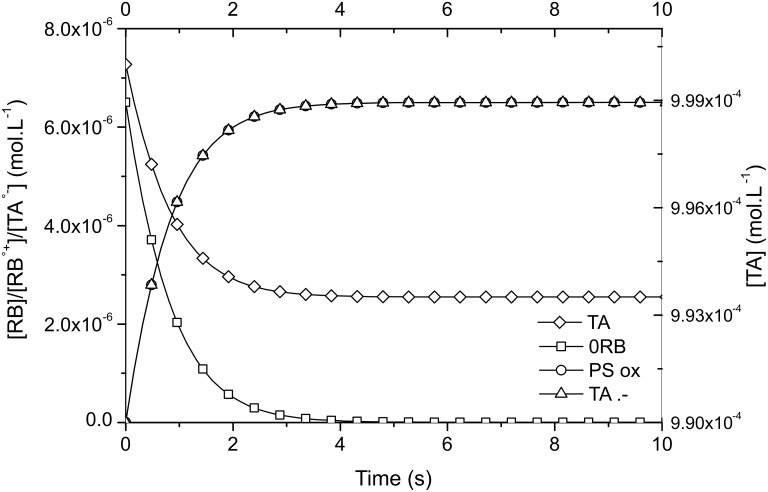
Evolution of RB, RB^•+^, TA, and TA^•−^ concentration with time for RB/TA system.

The Type II system studied here contains only RB as photosensitizer and an electron acceptor TA (or donor EDB). Both RB/TA and RB/EDB systems were calculated. Figure 4 shows the changes in RB, RB^•+^, TA, and TA^•−^ concentrations for the former system.

Two important conclusions are to be outlined. First, the ground state of the dye is quickly bleached: in about 4 seconds it has completely disappeared. Second: the final TA^•−^ concentration, i.e., the maximum number of initiating radicals produced is equal to the initial ground state RB concentration, i.e., 6.50·10^−6^ M. The final triazine concentration remains high because only 6.50·10^−6^ M of TA have reacted with the dye RB. The same conclusion can be drawn for the RB/EDB computation but with slower rates due to lower reactivity (i.e., *k*_q/D_). As a conclusion, in Type II PIS the limiting component for radical generation is the concentration of the dye.

### Simulation of photocyclic initiating system

**Figure 5:**
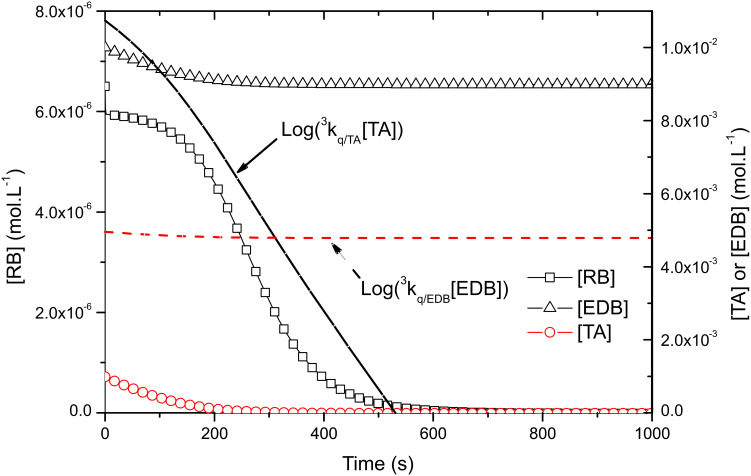
Evolution of RB, TA and EDB concentrations in the photocyclic system. The logarithm of oxidative and reductive pseudo first order reaction rates are given in plain and dashed lines, respectively.

In the photocyclic system, the initial pseudo first-order reaction rates are equal to ^3^*k*_q/A_[TA]_0_ = 1.7·10^5^ s^−1^ for TA (acceptor A) and ^3^*k*_q/D_[EDB]_0_ = 4.5·10^1^ s^−1^ for EDB (donor D). This means that the oxidative photocycle preferentially occurs, at least until ^3^*k*_q/A_[TA] > ^3^*k*_q/D_[EDB]. The plot of Log(^3^*k*_q/A_[TA]) and Log(^3^*k*_q/D_[EDB]) on Figure 5 show that it is the case for the first 310 sec of the reaction: during this period one can assume that the oxidative pathway is the main reaction.

The evolution of [RB], [TA] and [EDB] displayed on Figure 5 is also very interesting. Two major differences with Type II PIS should be outlined: first, the acceptor TA is totally consumed as its concentration falls to zero. Second, the ground state RB concentration, presents only a slight decrease during 200 s, i.e., as long as enough acceptor TA is present in the solution. This first part of the cycle should be explained by the reduction of the oxidized dye RB^•+^, by EDB leading to a recovery of the dye. As soon as no more TA is available (around 250 s) [RB] lowers faster to zero. In this second part the photocatalytic system is reduced to a conventional Type II system, where the dye is consumed during its reaction with the excess of amine EDB. If the TA acceptor is completely consumed this is not the case for the amine present in excess, only one TA equivalent is lost during the first part of the cycle, and [EDB] reduces to 0.9·10^−2^ M. During the second part of the reaction, a more tiny 6.5·10^−6^ M is consumed (i.e. the initial RB concentration, cf. type II PIS) leading to a final EDB concentration of 8.9935·10^−3^ M.

**Figure 6:**
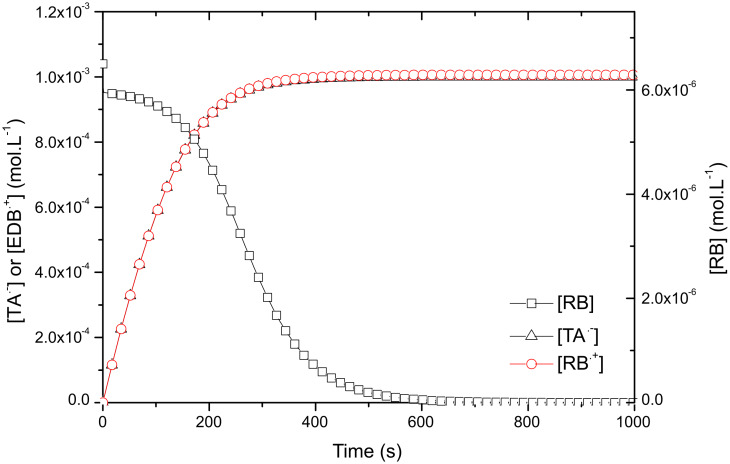
Evolution of radical concentrations TA^•−^ and EDB^•+^ together with [RB] in photocatalytic system.

On Figure 6, the evolution of EDB^•+^ and TA^•−^ concentrations are displayed together with RB. In the first part of the cycle (before 300 s) both radical curves are similar. Then, as soon as TA is consumed, no more TA^•−^ is produced and a final concentration of 1·10^−3^ M is reached, i.e. the initial TA concentration. At this stage the EDB^•+^ also reaches 1·10^−3^ M. During this second part, the reaction of excess EBD with RB leads to a tiny more 6.5·10^−6^ M for EDB^•+^ and to the bleaching of the dye. Thus, the computed final total radical concentration is formally equal to 2.0065·10^−3^ M.

It is worth emphasizing the important points and advantages of photocyclic systems:

1. the dye concentration is kept high for 250 s, this means that the absorbance of the solution is high, leading to a very high photon absorption of the solution during the first part of the cycle;

2. the final radical concentration is very high: 2.0065·10^−3^ M i.e. 150 times the concentration obtained in Type II PIS;

3. the limiting component is no more the dye, but the co-initiator of lowest concentration, in the present case the acceptor TA.

All this phenomena explain the synergistic effect observed in some free radical polymerization.

### Radical and photolysis quantum yields

As for the Type II systems, in order to quantify the catalytic behavior of the dye, the quantum yield of dye photolysis (Φ_photolysis_) in PCIS was determined. The radical quantum yield is defined by the ratio of the number of produced radical to the total number of absorbed photons (*N*_abs_). It could be expressed by the following equation:

[4]
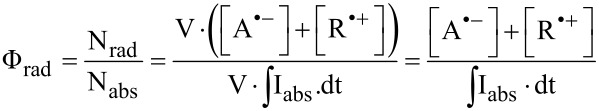


The calculated quantum yield for Type II RB/TA and photocyclic RB/TA/EDB are given in Table 3.

**Table 3:** Calculated photolysis and radical quantum yields of the Type II and the PCIS.

	Type II RB/TA	PCIS RB/TA/EDB

Φphotolysis	0.475	1.55·10^−3^
Φrad	0.475	0.478

In Type II PIS both quantum yields are equal because the amount of radicals formed is equal to the amount of dye photolysed: as a consequence, only about two photons are needed to bleach one dye molecule, corresponding to a very low turnover number. Moreover we can see that the system has an average efficiency: according to Φ_rad_ one photon on two is lost for radical generation. For photocyclic system, the picture is somehow different: the photolysis quantum yield falls to 1.55·10^−3^, meaning that more than 600 photons are needed to bleach one dye molecule: the turnover number becomes very high for the PCIS selected for the computation. However, if the photocatalytic behavior is very good, the radical quantum yield present an average value around 0.478, especially if one keep in mind that it could rise up to two, theoretically. But this is in line with the low rate constant of electron transfer of RB excited singlet and triplet states: the wasting photophysical cycle is comparatively too fast, preventing any efficient chemical energy escape from the photophysical cycle

However one can see from the experimental results that the consumption of the dye is longer than awaited for the Type II PIS, while it is shorter for the photocatalytic system. This is confirmed by comparing the experimental photolysis quantum yields to the computed values: the experimental photolysis quantum yield is higher and the turn over number of the real photocatalytic system RB/TA/EDB is lower than expected, while the opposite stands for RB/TA. This should be due to the fact that the bleaching of the dye is not only a direct consequence of the electron transfer reaction but could be due also to secondary reactions between the radicals and the dye related intermediates. As a consequence, the experimental efficiency of the photocycle is lower than the simulated one. This is clear from the shape of the two experimental curves where the consumption of RB in RB/TA is initially fast and then decreases slower while the opposite stands for the RB/TA/EDB: the consumption increases due to an increase of radical in the solution.

## Conclusion

In this paper experimental and full mechanistic studies of Type II PIS and 3-cpt photocatalytic systems were presented and compared. Some advantages that PCIS bear over classical Type II systems are: dye regeneration with high possible turnover (600), high radical production (>10^−3^ M). But these studies also reveal the importance of component choice: great care must be taken in order to build photocatalytic radical generator by combination of three compounds. Especially a simple thermodynamic approach should help to select the candidates as a starting point. If the present paper was focused on PCIS application for free radical photopolymerization, it should be noted that the same type of system bearing the same underlying kinetics and mechanisms are used in the field of photocatalytic water reduction.

## Experimental

Redox potentials were measured by cyclic voltammetry using a potentiostat (Princeton Applied Research 263A) at a scan rate of 1 V/s in acetonitrile, with platinum as both working and auxiliary electrodes, and a saturated calomel reference electrode (KCl in methanol). Measurements were performed in acetonitrile using 0.1 M of tetrabutylammonium hexafluorophosphate (Aldrich) as supporting electrolyte. The samples were bubbled with argon for 20 minutes prior to the analysis. Ferrocene was used as standard [60].

Steady sate UV–vis spectra were obtained on a Varian Cary 4000 UV–vis double beam spectrophotometer in 1 cm path quartz UV grade cell.

Laser flash photolysis experiments (LFP) were carried out exciting at 532 nm with a nanosecond Nd-YAG laser (Powerlite 9010, Continuum), operating at 10 Hz. The transient absorption analysis system (LP900, Edinburgh Instruments) uses a 450 W pulsed Xe arc lamp, a Czerny–Turner monochromator, a fast photomultiplier, and a transient digitizer (TDS 340, Tektronix) [61]. The instrumental response was about 7 ns. The observation wavelength is indicated in each case. Experiments were performed in acetonitrile under Ar bubbling.

A FluoroMax-4 (Horiba, Jobin-Yvon) spectrofluorometer coupled with a Time-Correlated Single-Photon Counting (TCSPC) accessory was used to measure the steady-state fluorescence spectra and singlet excited state lifetimes. NanoLEDs were used as pulsed excitation source leading to a time resolution of around 200 ps. The measurements were performed in acetonitrile solutions under argon bubbling at room temperature. The quenching rate constants *k*_q_ of the excited states were obtained according to the Stern–Volmer analysis where the reciprocal lifetime is plotted as a function of quencher concentration: [57].

Chemicals: Rose Bengal extra (RB) and ethyl 4-(dimethylamino)benzoate (EDB) were obtained from Aldrich, 2-(4-methoxyphenyl)-4,6-bis(trichloromethyl)-1,3,5-triazine (TA) was gifts from PCAS (France). Their chemical structures are given in Scheme 1.
